# Exploring Chinese herbal medicine for ischemic stroke: insights into microglia and signaling pathways

**DOI:** 10.3389/fphar.2024.1333006

**Published:** 2024-01-22

**Authors:** Wenjing Zhang, Haoqun Xu, Chong Li, Bingbing Han, Yimin Zhang

**Affiliations:** College of Traditional Chinese Medicine, Shandong University of Traditional Chinese Medicine, Jinan, Shandong, China

**Keywords:** natural compounds in traditional Chinese herbal medicines, microglia, ischemic stroke, signaling pathway, inflammatory response, drug-delivery systems

## Abstract

Ischemic stroke is a prevalent clinical condition affecting the central nervous system, characterized by a high mortality and disability rate. Its incidence is progressively rising, particularly among younger individuals, posing a significant threat to human well-being. The activation and polarization of microglia, leading to pro-inflammatory and anti-inflammatory responses, are widely recognized as pivotal factors in the pathogenesis of cerebral ischemia and reperfusion injury. Traditional Chinese herbal medicines (TCHMs) boasts a rich historical background, notable efficacy, and minimal adverse effects. It exerts its effects by modulating microglia activation and polarization, suppressing inflammatory responses, and ameliorating nerve injury through the mediation of microglia and various associated pathways (such as NF-κB signaling pathway, Toll-like signaling pathway, Notch signaling pathway, AMPK signaling pathway, MAPK signaling pathway, among others). Consequently, this article focuses on microglia as a therapeutic target, reviewing relevant pathway of literature on TCHMs to mitigate neuroinflammation and mediate IS injury, while also exploring research on drug delivery of TCHMs. The ultimate goal is to provide new insights that can contribute to the clinical management of IS using TCHMs.

## Graphical Abstract

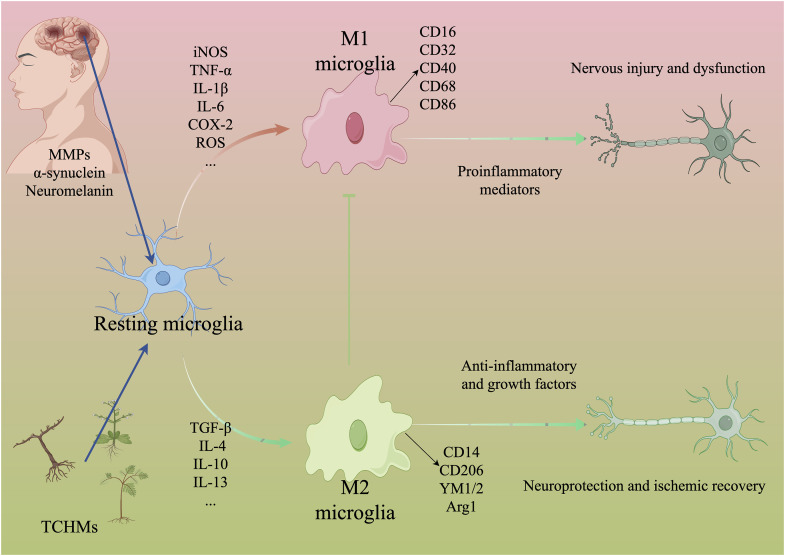

## 1 Introduction

Ischemic stroke (IS) is a prevalent central nervous system disorder characterized by elevated mortality and disability rates, posing a significant threat to human wellbeing. Currently, thrombolysis stands as the primary clinical intervention (ANTTILA et al., 2017). Nevertheless, due to the limitation of thrombus autolysis or the time window of thrombolytic drugs, leading to subsequent neuronal damage caused by cerebral ischemia-reperfusion injury. The pathogenesis of cerebral ischemia and reperfusion injury is intricate in nature. Interfering with the activation of microglia or its subsequent overactivation is believed to play a crucial role in the pathogenesis of cerebral ischemia and reperfusion injury (LIU et al., 2017). As a resident immune cell in the brain, microglia promptly initiate phagocytosis of pathogens and tissue repair following injury stimulation. Nevertheless, the excessive activation of microglia results in the release of numerous pro-inflammatory factors and neurotoxic substances, thereby triggering a cascade amplification of the inflammatory response and contributing to the development of cerebral ischemia and reperfusion injury (LIU et al., 2017). The modulation of microglia overactivation and polarization phenotype, as well as the inhibition of the inflammatory cascade, present a promising avenue for the treatment of cerebral ischemic injury. Traditional Chinese herbal medicines (TCHMs) offer notable advantages such as enhanced safety, minimal side effects, and proven efficacy. Additionally, their multi-target and multi-channel characteristics make them suitable for addressing the intricate pathological mechanisms underlying cerebral ischemia and reperfusion injury. Consequently, the development of TCHMs specifically targeting microglia mediated IS has emerged as a pressing issue in need of resolution. Interfering with the activation of microglia or its subsequent overactivation is believed to play a crucial role in the pathogenesis of cerebral ischemia and reperfusion injury. As a resident immune cell in the brain, microglia promptly initiate phagocytosis of pathogens and tissue repair following injury stimulation. Nevertheless, the excessive activation of microglia results in the release of numerous pro-inflammatory factors and neurotoxic substances, thereby triggering a cascade amplification of the inflammatory response and contributing to the development of cerebral ischemia and reperfusion injury.

This paper aims to discuss the biological characteristics of microglia, their relationship with cerebral ischemia injury, the associated inflammatory signaling pathways, and the research on drug delivery of TCHMs. The objective is to offer insights and references for the advancement of novel drugs for clinical treatment of IS.

## 2 Biological characteristics of microglia

In the late 19th century, the identification and characterization of microglia, also referred to as ‘rod cells’, revealed their reactive, migratory, proliferative, and phagocytic properties within the brain (NAYAK et al., 2014). Santiago Ramon y Cajal distinguished microglia as one of the three primary components of the nervous system, distinct from neurons and glial cells, originating from the mesoderm. Subsequently, Del Rio-Hortega officially designated them as microglia (LOW and GINHOUX, 2018). Extensive research has demonstrated the widespread distribution of microglia throughout the brain and spinal cord, constituting approximately 5%–10% of all brain cells (HICKMAN et al., 2018). Microglia exhibit morphological characteristics similar to macrophages, and the presence of the macrophage marker integrin CD11b has been observed in both human and mouse microglia. Consequently, microglia have been identified as macrophages associated with the brain (CUADROS et al., 2022). Furthermore, genetic investigations have demonstrated that the absence of the transcription factor PU.1, crucial for myeloid cell development, impedes the maturation of microglia (PERRY et al., 1985). This finding suggests that microglia originate from hematopoietic cells in the bone marrow and are generated by progenitor cells in the embryonic yolk sac during the early stages of embryogenesis, and that it works with the blood-brain barrier (BBB) to maintain brain homeostasis (LEAVY, 2010; AGUZZI et al., 2013; CUADROS et al., 2022). Under normal physiological conditions, microglia remain in a quiescent state and exhibit a branched morphology. They navigate through the brain parenchyma by incessantly oscillating their slender protrusions on the surface, thereby surveilling alterations in the microenvironment and upholding the homeostasis of the central nervous system (ZHANG, 2019). Conversely, in pathological circumstances, microglia can undergo prompt activation, proliferation, and subsequent migration towards the site of injury. This leads to an enlargement of the cell body, reduction in process length, and a transition from a branched to an amoeboid morphology (LIU et al., 2019). In various microenvironments, activated microglia have the ability to undergo polarization into two distinct phenotypes known as ‘classically activated’ M1 and ‘alternatively activated’ M2 (LIU et al., 2018a). M1 microglia exhibit a pro-inflammatory function by enhancing phagocytosis and pathogen elimination, as well as promoting tissue repair. However, excessive activation of M1 microglia can lead to the release of harmful substances such as interleukin (IL), tumor necrosis factor (TNF-α), inducible nitric oxide synthase (iNOS), and reactive oxygen species (ROS). This release triggers a series of inflammatory cascades, exacerbating the extent of damage (LIU et al., 2017; LOW and GINHOUX, 2018). M2 macrophages exert anti-inflammatory effects through the secretion of anti-inflammatory mediators (IL-4, IL-10, IL-13) and neurotrophic factors, thereby mitigating inflammation, safeguarding neurons, and facilitating tissue regeneration (LOW and GINHOUX, 2018; XUE et al., 2021).

## 3 Relationship between microglia and ischemic stroke

Following cerebral ischemia injury, dying neurons release matrix metalloproteinases, α-synuclein, and neuromelanin to attract and stimulate microglia, which then phagocytose damaged neurons and debris, thereby mitigating brain tissue damage (LI et al., 2021a). However, in the case of cerebral ischemia and reperfusion injury, the persistent activation of microglia within the brain not only triggers a pronounced inflammatory response but also initiates a cascade of reactions including oxidative stress, endoplasmic reticulum stress, and apoptosis (ZARRUK et al., 2018; TIAN and MAO, 2022). Clinical data demonstrate that the activation of microglia is observable throughout all stages of IS development (GULYÁS et al., 2012). Animal models reveal that microglial cells undergo dynamic changes temporally and spatially as the disease advances, and their alterations are associated with the severity of IS (WANG et al., 2023a). Importantly, it should be emphasized that microglial activation and polarization in IS possess a dual nature, encompassing both neuroprotective properties and the capacity to inflict nerve damage. Previous research has demonstrated that during the subacute phase of cerebral ischemia, M1 microglia, which are responsible for initiating the inflammatory response, become activated and contribute to the progression of inflammation (WANG et al., 2023b). Conversely, the release of M2 microglia during ischemic recovery has been shown to play a role in stroke repair (LIU X. et al., 2018). Consequently, modulating the polarization of microglia towards M2 and inhibiting their polarization towards M1 during cerebral ischemia and reperfusion injury may be beneficial in mitigating the damaging effects of inflammation (JIANG et al., 2021). By maintaining a balance between pro-inflammatory and anti-inflammatory responses through the regulation of M1/M2 microglia polarization in the brain, saving cerebral ischemia and reperfusion injury and helping tissue repair and remodeling are particularly important treatment methods. In addition, studies have also found that the number of activated microglia is positively correlated with the degree of cerebral ischemia and reperfusion injury, especially in the cerebral ischemic penumbra, and its excessive activation further leads to the aggravation of brain edema (ZARRUK et al., 2018). The activation and polarization of microglia, along with the resulting inflammatory response, are regarded as pivotal factors in the pathogenesis of cerebral ischemia and reperfusion injury. Consequently, the activation of microglia to induce a neuroinflammatory response is also being explored as a potential therapeutic approach.

## 4 The mechanism of TCMHs intervention in microglia and its related signaling pathways in the treatment of ischemic stroke

### 4.1 1NF-κB signaling pathway

The nuclear factor-kappa B (NF-κB) pathway is known to have a significant impact on the occurrence of cerebral ischemic injury, as it is capable of inducing microglia polarization and initiating an inflammatory response (DORRINGTON and FRASER, 2019). In pathological circumstances, the inhibitor of NF-κB (IκB) undergoes phosphorylation and degradation, leading to the release of the NF-κB subunit p65 into the nucleus, thereby activating microglia and initiating an inflammatory response (DORRINGTON and FRASER, 2019; HE et al., 2019). Furthermore, cerebral ischemia-induced elevation of ROScan stimulate the activation of NF-κB, thereby facilitating the inflammatory response. Notably, microglia exhibit a high expression of NADPH oxidase (respiratory burst oxidase homologue, Rboh), which serves as the primary source of ROS. Consequently, inhibition of the NF-κB signaling pathway, reduction in Rboh activity, and subsequent decrease in ROS production within microglia can be achieved and the downregulation of proinflammatory mediators is regarded as a viable therapeutic strategy for the treatment of cerebral ischemia (NAM et al., 2010; WU et al., 2018; MI et al., 2021).

**FIGURE 1:**
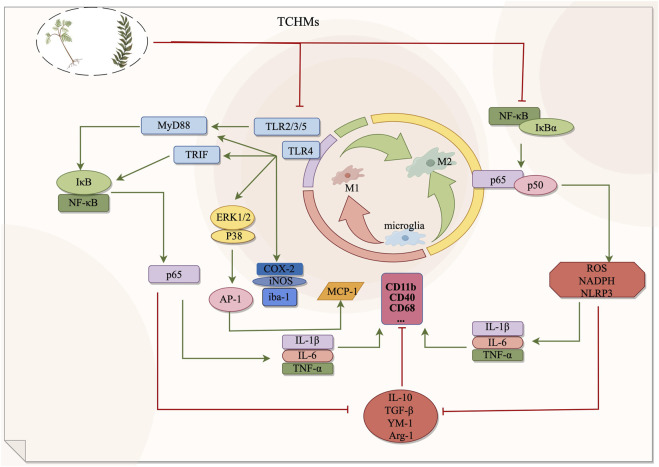
The NF-κB signaling pathway and Toll signaling pathway exert regulatory effects on microglia during the management of ischemic stroke. By suppressing microglial activation, these pathways can effectively impede the transition from microglia to M1 phenotype or facilitate the polarization from M1 to M2 phenotype, consequently safeguarding cerebral nerves and diminishing the secretion of inflammatory mediators. In this context, red signifies inhibition, while green signifies promotion.

*Salvianolic acid A* (*SalA)*, an active component of *Salvia miltiorrhiza*, exhibits notable antioxidant, anti-inflammatory, and anti-thrombotic properties, rendering it efficacious in treating various central nervous system disorders (CHOI et al., 2017; ZHANG et al., 2018a; FENG et al., 2020). Research indicates that SalA hinders the nuclear translocation of NF-κB, diminishes the phosphorylation level of IκBα, suppresses NO production and iNOS protein expression in lipopolysaccharide (LPS)-induced mouse small glioma cell line (BV2), downregulates TNF-α, IL-1β, and IL-6, ameliorates nerve function impairment, and mitigates inflammation (HUANG et al., 2023). *Anisalcohol*, *Kellerin*, *Neocryptotanshinone*, and other TCHMs have been found to have the ability to interfere with the NF-κB pathway. This interference leads to a reversal of the inflammatory response induced by microglia polarization, achieved by increasing the expression of M2 markers of microglia (CD14, CD206, YM1/2, Arg1) or decreasing the expression of M1 markers of microglia (CD16, CD32, CD40, CD68, CD86, iNOS). Consequently, the release of pro-inflammatory factors such as TNF-α, interleukin-1β (IL-1β), and interleukin-6 (IL-6) is reduced, while the expression of anti-inflammatory factors such as TGF-β, IL-4, and IL-10 is increased. This modulation of cytokine expression ultimately leads to an improvement in brain tissue injury. Further details regarding the specific mechanisms involved can be found in Table 1 and Figure 1.

**TABLE 1:** Mechanism of intervention of microglia and NF-κB signaling pathway mediated by natural plant drugs in ischemic stroke.

Type	Name	Experimental model	Effect↑↓	Reference
Organic acids	Kellerin	MCAO rat model (SD) and LPS cell model (BV2)	↓NF-κB-p65, p-IκBα, ROS, NADPH and (CD11b)	MI et al. (2021)
Flavonoids	Kaempferol	I/R rat model (SD)	↓NF-κB-p65, iNOS, COX-2, IL-6, IL-1β and TNF-α	LI et al. (2019)
	Baicalein	OGD/R cell model (SH-SY5Y) and MCAO rat model (SD)	↓NF-κB, TNF-α, IL-1β and IL-6, ↑IL-10	YUAN et al. (2020)
	Anthocyanins	MCAO/R rat model (SD)	↓NF-κB-p65, NF-κB-p50, IκB, NLRP3, IL-1β, TNF-α and IL-6	PAN et al. (2018)
	Scutellarin	MCAO rat model (SD) and LPS cell model (BV2)	↓NF-κB, iNOS, IL-1β and TNF-α	YUAN et al. (2014)
	Genistein-3′-sodium sulfonate	tMCAO rat model (SD) and LPS cell model (BV2)	↓NF-κB-p65, p-IκB, p-IKK, CD11b, CD40, CD68, TNF-α and IL-1β	LIU et al. (2021a)
Diketones	Curcumin	OGD/R cell model (BMECs)	↓NF-κB-p65, IκB and IL-1β	DONG et al. (2014)
Terpenoids	Neocryptotanshinone	MCAO mice model (C57BL/6) and LPS/IFN-γ cell model (BV2)	↓NF-κB-p65, NF-κB -p-P50, IL-6, IL-1β, TNF-α and CD11 ↑CD14	ZHAI et al. (2023)
	Ginsenoside Rd	MCAO rat model (SD) and OGD cell model (BV2)	↓NF-κB-p65, IκBα, IL-1β, IL-6, IL-18, TNF-α, IFN-γ and Iba-1	ZHANG et al. (2016)
Phenols	Anisalcohol	LPS cell model (BV2)	↓NF-κB-p65, p-p65, TNF-α, IL-1β and IL-6, CD16/32 ↑TGF-β, IL-10 and CD206	XIANG et al. (2018)
	6-Shogaol	2VO mice model (C57BL/6) and LPS cell model (BV2)	↓NF-κB-p65, p-IκB, iNOS, COX-2, IL-1β and TNF-α	HA et al. (2012)
Glycosides	Salidroside	LPS cell model (BV2)	↓NF-κB-p65, IκBα and IL-8	HU et al. (2014)
Phenylpropanoids	α-Asarone	OGD/R cell model (BV2)	↓NF-κB-p65, NLRP3, TNF-α ↑TGF-β, IL-10 and IL-4	XU et al. (2022)

### 4.2 Toll signaling pathway

Toll-like receptors (TLRs) are a class of transmembrane proteins with type I topology, comprising three distinct components: extracellular regions responsible for the recognition of extracellular pathogens and signals of tissue damage, transmembrane regions, and intracellular regions involved in the transmission of downstream signals (CHEN et al., 2023a). The TLR family encompasses TLR1-10, and the intracellular region of TLR initiates the activation of signaling pathways by interacting with adaptor molecules that possess Toll/interleukin-1 receptor (TIR) domains, such as MyD88, TIRAP, TRIF, and TRAM (ZHANG et al., 2020a). TLR4 represents a pivotal receptor involved in the activation and functioning of microglia, which have been observed to undergo activation subsequent to ischemic brain injury, thereby leading to an upregulation in the expression of TLR4. It is a crucial receptor involved in the activation and functioning of microglia. Research has demonstrated that microglia become activated following ischemic brain injury, leading to an upregulation of TLR4 expression. This activation triggers the intracellular MyD88-dependent signal transduction pathway, resulting in the rapid phosphorylation of IκB and subsequent dissociation of NF-κB, allowing it to enter the nucleus. Additionally, TLR4 can collaborate with activator protein 1 (AP-1), which becomes activated through the phosphorylation of p38 and ERK1/2 (CHEN et al., 2023a). This collaboration promotes the release of pro-inflammatory cytokines such as TNF-α, IL-1, IL-6, IL-8, and IL-12, leading to a cascade of inflammatory responses (WANG et al., 2016; ZHANG et al., 2020a; CHEN HD. et al., 2023). Therefore, the loss of TLR4 function may play a neuroprotective effect (CHEN HD. et al., 2023).

*Gardenia*, a traditional Chinese medicine, is utilized for medicinal and dietary purposes. The active component, *Geniposide (GEN)*, has been scientifically demonstrated to possess anti-inflammatory, antihypertensive, and neuroprotective properties (WU et al., 2019). The researchers employed various concentrations of gardennia side to disrupt hypoxic/reoxygenated microglia. They discovered that a concentration of 500 μmol/L of GEN effectively reduced the expression of TLR4, MyD88, p-IκB, NF-κB, p-ERK1/2, and p38 proteins in the treatment group, which exhibited significant differences compared to the model group. These findings suggest that GEN possesses the ability to inhibit hypoxia/reoxygenation and activate microglia, thereby exerting an anti-inflammatory effect through the downregulation of the TLR4-dependent pathway of MyD88. Furthermore, it is proposed that gardenin can facilitate the recovery of cerebral ischemia (HOU et al., 2011). *Baicalin*, an active constituent of *scutellaria baicalensis*, exhibits similar properties to gardeniin by diminishing microglial activity and exerting a neuroprotective effect in the brain through the inhibition of TLR4 pathway and downstream protein expression (HOU et al., 2012). *Polygalasaponin F (PGSF)*, a *triterpenoid saponin* compound derived from melon *seed gold of Polygala* (SUN et al., 2022), was utilized by Shi et al. (SHI et al., 2017) to intervene lipid-induced BV-2 microglia in order to simulate a neuroinflammation model following cerebral ischemia. The study revealed that PGSF effectively counteracted the upregulation of Toll-like receptor 4 (TLR4) in microglia, downregulated the expression of nitric oxide synthase (iNOS) and cyclocycesterase-2 (COX-2) induced by cellular inflammatory proteases, ameliorated the over-activation of microglia and the production of neurotoxic factors, and mitigated nerve cell injury. Furthermore, the downregulation of microglial overactivation can be achieved through the inhibition of the TLR4 signaling pathway by *curcumin*, *anthocyanin*, *paeoniflorin*, and *quercetin*. This intervention leads to a decrease in the expression of ionized calcium binding adaptor molecule 1 (Iba-1), a recognized marker of microglia, thereby improving nerve injury. For a comprehensive understanding of the underlying mechanisms, please refer to Table 2; Figure 1.

**TABLE 2:** Mechanism of intervention of microglia and toll-like signaling pathway mediated by natural plant drugs in ischemic stroke.

Type	Name	Experimental model	Effect↑↓	Reference
Organic acids	Salvianolic acid	MCAO rat model (SD) and LPS cell model (BV2)	↓TLR4, NF-κB, IL-1β, IL-6 and Iba-1	ZHUANG et al. (2017)
		MCAO mice model (WT) LPS/IFN-γ cell model (BV2)	↓TLR4, MyD88, NF-κB and Iba-1	SHEN et al. (2022)
Flavonoids	Baicalin	OGD cell model (BV2)	↓TLR4, MyD88, p-IκB, NF-κB, p-ERK1/2 and p38	HOU et al. (2012)
	Baicalein	OGD/R cell model (primary microglia)	↓TLR2/4, MyD88, NF-κB	LI et al. (2012), YUAN et al. (2020)
	Anthocyanins extracts	I/R mice model (ICR)	↓TLR4-NF-κB, TNF-α and IL-18	CUI et al. (2018)
	Quercetin	OGD cell model (BV2)	↓TLR4, MyD88, p-IκB, NF-κB, IL-1β, IL-6, TNF-α and iba1	LE et al. (2020)
	Paeoniflorin	H/I rat model (SD)	↓TLR4, NF-κB, IL-1β and TNF-α	YANG et al. (2021)
	Luteolin	pMCAO rat model (SD) and MCAO rat model (SD)	↓TLR4, TLR5, NF-κB and iNOS	QIAO et al. (2012)
	Nobiletin	I/R rat model (SD)	↓TLR4, NF-κB-p65, NO, IκB-α, IKK-βIL-1β, TNF-α and IL-6	ZHENG et al. (2017)
	Orientin	I/R rat model (SD)	↓TLR4, NF-κB, IL-1β, TNF-α and IL-6 ↑ SOD	WANG et al. (2017a)
	Schaftoside	OGD/R cell model (BV2)	↓TLR4, MyD88, IL-1β, TNF-α, IL-6	ZHOU et al. (2019)
Diketones	Curcumin	OGD cell model (BV2) LPS cell model (Cortical neuronal)	↓TLR4, MyD88, NF-κB-p65, IκB-α, MCP-1, IL-1β, TNF-α, IL-6 and CD11	TU et al. (2014), ZHU et al. (2014)
Terpenoids	β-Caryophyllene	I/R mice model (C57BL/6J) and LPS cell model (primary microglia)	↓TLR4, TRIF, My D88, iNOS, TNF-α and CD68 ↑ TGF-β, YM-1, Arg-1 and CD206	TIAN et al. (2019)
	Senkyunolide I	OGD cell model (BV2)	↓TLR4, IKKα/β, NF-κB-p65 and iNOS ↑Hsp70	HU et al. (2016)
	Ginsenoside Rg1	MCAO rat model (SD) and OGD/R cell model (primary microglia)	↓TLR4, My D88, NF-κB, TRIF, IRF-3 L-1β, IL-6, TNF-α and Iba-1	GUAN et al. (2023)
Glycosides	Gypenoside	MCAO mice model (C57BL/6) and OGD cell model (BV2)	↓TLR4, NF-κB-p65, iNOS and IL-6 ↑CD206, TGF-β and Arg1	XIA et al. (2023)
	Salidroside	MCAO rat model (SD) and OGD cell model (BV2)	↓TLR4, My D88, NF-κB, NLRP3, IL-1β, IL-18 and Iba-1	LIU et al. (2021b)
Phenols	6-Shogaol	2VO mice model (C57BL/6) and LPS cell model (BV2)	↓TLR3/4, MyD88, TRIF, iNOS, IL-1β and TNF-α	HA et al. (2012)
Alkaloid	Berberine	OGD cell model (BV2)	↓TLR4, iNOS and TNF-α	TAO et al. (2019)
Phenylpropanoids	Ferulic acid	I/R rat model (SD)I/R cell model (PC12)	↓ TLR4, MyD88, Caspase-3, Bax, MDA. ↑ Bcl-2, SOD	REN et al. (2017)

### 4.3 Notch signaling pathway

The Notch signaling pathway consists of Notch receptors, Notch ligands, and effector molecules. Extensive research has demonstrated that the Notch signaling pathway plays a crucial role not only in the regulation of neurodevelopment but also in the pathogenesis of IS (WANG JJ. et al., 2019). Previous studies have demonstrated that the Notch signaling pathway becomes activated following cerebral ischemia. This activation occurs when the receptor of the pathway binds to ligands, resulting in the recruitment of the intracellular segment of the Notch receptor (NICD) (ZANOTTI and CANALIS, 2016). After being cleaved twice, the NICD enters the nucleus and binds to effector molecules, thereby regulating the transformation of microglia into the M1 phenotype. This process also promotes the nuclear shift of NF-κB and enhances the release of inflammatory mediators, ultimately accelerating the progression of IS (LI QQ. et al., 2021; LI et al., 2022). Furthermore, Guo et al. (GUO Z. et al., 2021) have discovered that Notch can activate microglia by mediating the ligand Jagged1, leading to the secretion of pro-inflammatory cytokines.

**FIGURE 2:**
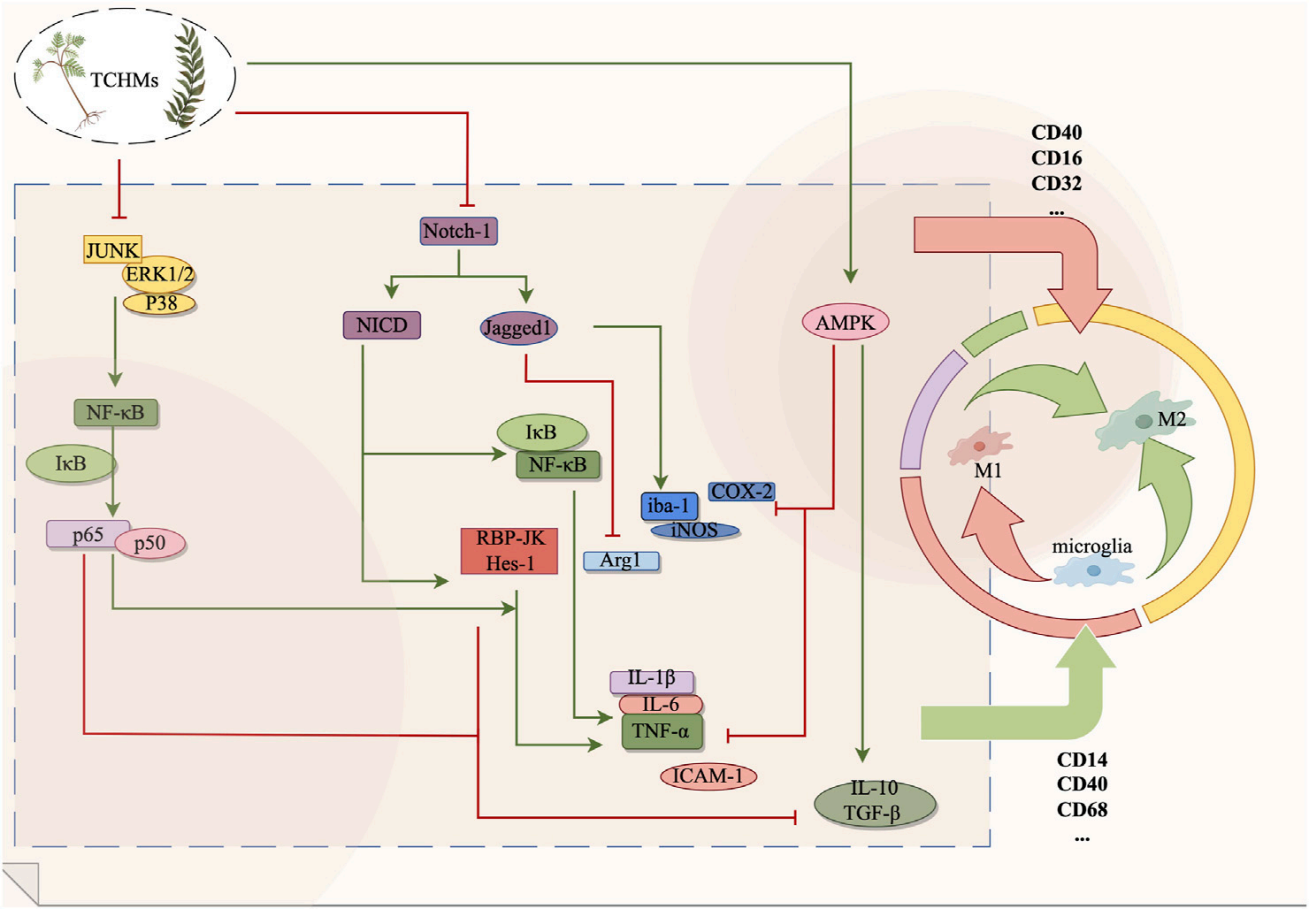
The Notch signaling pathway, AMPK signaling pathway and MAPK signaling pathway exert regulatory effects on microglia during the management of ischemic stroke. By suppressing microglial activation, these pathways can effectively impede the transition from microglia to M1 phenotype or facilitate the polarization from M1 to M2 phenotype, consequently safeguarding cerebral nerves and diminishing the secretion of inflammatory mediators. In this context, red signifies inhibition, while green signifies promotion.

*Curcumin(Cur)*, a potent compound derived from the subterranean rhizome of turmeric, possesses notable anticoagulant, anti-inflammatory, and neuroprotective properties (SHAHRAJABIAN and SUN, 2023). In their study, Ye et al. (Ye et al., 2021) observed that curcumin effectively suppresses the expression of Notch-1, curtails the excessive proliferation of microglia, and concurrently diminishes the levels of TNF-α and IL-1β in rats. Consequently, curcumin aids in preserving the normal neural function of rats experiencing cerebral ischemia-reperfusion. Numerous studies have demonstrated the neuroprotective effects of *Gastrodin*, a *phenolic glycoside* compound derived from *Gastrodia elata*, in the context of ischemic brain injury (XIAO et al., 2021). Previous research has demonstrated that the inhibition of the activation of the Notch signaling pathway can effectively decrease the recruitment of NICD. Consequently, this downregulates the expression of downstream recombining binding protein suppressor of hairless (RBP-JK), the transcription factor hairy and enhancer of split-1 (Hes-1), and TNF-α. This regulatory mechanism ultimately contributes to the amelioration of brain tissue damage resulting from the release of inflammatory factors (GUO et al., 2021b; YANG et al., 2023). *Vinpocetine (Vin)* is an *alkaloid* compound derived from *Vinca minor L*, known for its neuroprotective properties against cerebral ischemia-induced neuronal damage in rats (ZHANG et al., 2018b). The experiment demonstrated that the administration of Vin to cerebral ischemia rats resulted in a decrease in the protein expressions of Notch-1, NICD, and Jagged-1 in the hippocampus. At the same time, the presence of Iba-1 was observed, and the immunofluorescence analysis revealed a significant reduction in the number of M1-type microglia marker Iba-1/iNOS. Conversely, the expression of the marker Iba-1/Arg1 in M2 microglia exhibited a significant increase. Furthermore, the levels of IL-1β and TNF-α decreased, while the levels of IL-10 increased (CHEN et al., 2022). Vin has been proposed to regulate the M1/M2 polarization of microglia through its mediation of the Notch signaling pathway, thereby inhibiting the inflammatory response and enhancing neuronal recovery following cerebral ischemia. Furthermore, baicalein has been demonstrated in both *in vivo* and *in vitro* experiments to reduce the expression of notch-1, NICD, and Hes-1 in microglia by inhibiting the Notch signaling pathway, thereby exerting a protective effect on brain tissue (YUAN et al., 2015; YUAN et al., 2016). In their study, Zhang et al. (ZHANG et al., 2020b) discovered that the administration of rhubarb resulted in a notable enhancement of Iba-1 positive cells within the ischemic penumbra region of MCAO rats. Additionally, rhubarb exhibited the ability to diminish the infarct area, suppress microglia activation, downregulate the Notch signaling pathway, and decrease the expression of neuroinflammatory mediators, including ICAM-1 and TNF-a (shown in Figure 2).

### 4.4 AMPK signaling pathway

AMP-activated protein kinase (AMPK) is widely recognized as a prominent sensor of cellular energy fluctuations and physiological stress, and it assumes a crucial function in maintaining cellular homeostasis (CAO et al., 2019). Prior research has elucidated the close association between AMPK and inflammatory signaling pathways, whereby AMPK activation impedes NF-κB activation and mitigates the release of inflammatory mediators (LIN et al., 2021). Then, the activation of the AMPK signaling pathway subsequent to cerebral ischemia effectively modulates microglial polarization and attenuates cerebral tissue injury (CHEN et al., 2023b).

*Z-ligustilide (LIG)*, an extract derived from Chinese herbs such as *Chuanxiong*, *Angelica*, and *ligusticum*, has been scientifically demonstrated to possess notable neuroprotective properties (WU et al., 2011). In a study conducted by Yu et al. (GAN et al., 2020), it was observed that the Phthalide derivative CD21 of LIG exhibited the ability to decrease the volume of cerebral infarction and mitigate neurological impairments, thereby functioning as a neuroprotective agent in cases of ischemic brain injury. This compound was found to activate the AMPK signaling pathway by elevating the levels of p-AMPK in both ischemic brain tissue and BV2 cells. Furthermore, it was observed to reduce the expression of CD86, IL-1β, and iNOS in BV-2 cells, while concurrently upregulating CD206, IL-10, and YM1/2. These effects were found to ameliorate neuroinflammation and facilitate the process of neural repair. The *compound 3C*, which is a derivative of *Balasubramide*, is an e*ight-member lactam* compound that has been isolated from Clausena lansium. Skeels, Multiple studies have demonstrated that the administration of balasubramide Derivative 3C leads to a significant upregulation of AMPK and its upstream target calmodulin-dependent protein kinase β (CaMKKβ), thereby activating the M2 polarization of microglia (WANG et al., 2018a). This activation subsequently results in a reduction in the release of proinflammatory factors such as TNF-α, IL-1β, IL-6, iNOS, and COX-2. Consequently, in a rat model of tMCAO, the infarct area was reduced, while in BV2 cells, the production of proinflammatory cytokines induced by LPS was improved and the administration of balasubramide Derivative 3C promoted sensorimotor recovery and inhibited the infiltration of inflammatory cells (WANG et al., 2018a; WANG et al., 2018b). *Sinomenine (SION)*, an *alkaloid* was found to inhibit the inflammatory body NLRP3 through the regulation of the AMPK signaling pathway in both the MCAO mouse model and OGD mixed glial cell model, It downregulated the expression of IL-1β, IL-6, IL-18, and TNF-α, and mitigated the excessive activation of microglia following cerebral ischemic injury, which shown in Figure 2 (QIU et al., 2016).

### 4.5 MAPK signaling pathway

The Mitogen-activated protein kinase family (MAPK) plays a pivotal role in mediating fundamental biological processes, transmitting external cell pressure signals, and actively participating in various cellular processes, including cell growth, proliferation, and differentiation (CHEN et al., 2019). The MAPK signal transduction pathways encompass c-Jun NH2 terminal kinases (JNK), p38 mitogen-activated protein kinase (p38MAPK), and extracellular signal-regulated kinase (ERK) (WANG et al., 2019b). Among these, p38MAPK holds significant importance as a member of the MAPK family involved in the regulation of inflammation (LIU et al., 2015a). Notably, the activation of p38MAPK can be induced by the release of inflammatory factors such as LPS, TNF-α, and IL. Above all, p38MAPK plays a role in the upregulation of NF-κB and the synthesis of various inflammatory mediators, including TNF-α, IL-6, IL-8, and COX-2, these actions ultimately impact the polarization of microglia (HWANG et al., 2018). Several studies have demonstrated the involvement of p38MAPK in the regulation of the pathological injury process associated with cerebral ischemia. Inhibiting the p38MAPK pathway has been shown to effectively decrease microglia activation, ameliorate cerebral ischemic inflammation, and provide neuroprotection (HWANG et al., 2018; LI et al., 2020a; ZHANG et al., 2022a). Furthermore, the activation of ERK1/2 has been observed to hinder the nuclear translocation of NF-κB, downregulate the expression of pro-inflammatory genes, suppress microglia activation and M1 polarization, and promote M2 polarization (MOHANRAJ et al., 2019; YU et al., 2022).

Previous studies have demonstrated the neuroprotective properties of *Cissus verticillata (CVE)* from *vitaceae* in the context of IS, with its mechanism of action involving the inhibition of the MAPK signaling pathway. Administration of 300 mg/kg CVE has been shown to effectively mitigate microglial activation and suppress the phosphorylation of JNK, ERK, and p38 and the expression of pro-inflammatory cytokines IL-1β, IL-6, and TNF-α was significantly reduced, leading to an amelioration of the inflammatory response and neuronal damage (WANG et al., 2022a). *4-Hydroxysesamin*, a *lignan constituent* of *tetrahydrofuran*, is obtained from Chinese herbs including *Rhizoma hydroxysesamin*, *Watkins*, and *camphor,* and has been found to possess anti-stroke properties (HADIPOUR et al., 2023). Research has demonstrated that 4-hydroxysesamin exerts its protective effects on cerebral ischemic tissue by down-regulating the expression of COX-2 and IL-6 through the inhibition of the p38MAPK signaling pathway (ZHANG et al., 2021). HA S K et al. (HA et al., 2008) discovered that *Apigenin* of *Flavonoids* has the ability to decrease the expression of p-JNK, p-ERK, and p-p38 in LPS-induced BV-2 microglia. Meanwhile, it can also suppress the production of inflammatory mediators such as NO and COX-2, thereby exhibiting anti-inflammatory effects through the inhibition of the MAPK signaling pathway. The involvement of Astragaloside IV, Total saponins of Panax japonicus, Scutellarin and other TCHMs in the regulation of microglia polarization and improvement of brain tissue inflammation through the mediation of the MAPK signaling pathway is elucidated in Table 3 and Figure 2.

**TABLE 3:** Mechanism of intervention of microglia and MAPK signaling pathway mediated by natural plant drugs in ischemic stroke.

Type	Name	Experimental model	Effect↑↓	Reference
Flavonoids	Scutellarin	MCAO rat model (SD) and LPS cell model (BV2)	↑p-ERK1/2, ↓p-p38 MAPK, p-JNK, iNOS, TNF-α and IL-1β	CHEN et al. (2020)
	Hesperetin	LPS cell model (BV2)	↓p -p38 MAPK, ERK, iNOS, IL-1β and IL-6	JO et al. (2019)
	puerarin	I/R cell model (PC12)	↑SOD	LI et al. (2023)
			↓p-JNK, JNK, caspase-3 and MAD	
	Poncirin	MCAO mice model (C57BL/6J) and LPS cell model (BV2)	↓JNK, ERK1/2 and IL-6	YANG et al. (2020)
	icariin	t MCAO rat model (SD)	↓ERK, NF-κB, IL-1β, TNF-α, Iba1 and CD40	YU et al. (2022)
			↑CD68	
	Luteolin	pMCAO rat model (SD) and MCAO rat model (SD)	↓p38 MAPK, iNOS	QIAO et al. (2012)
			↑ p-ERK1/2	
Diketones	Curcumin	OGD/R cell model (BMECs)	↓p38, JNK, MAPKs, and IL-1β	DONG et al. (2014)
Terpenoids	Tussilago farfara	t MCAO rat model (SD) and LPS cell model (BV2)	↓p-p38 MAPK, p-JNK, NF-κB, p-ERK1/2 iNOS, IL-1β and IL-6	HWANG et al. (2018)
	Thuja orientalis	t MCAO rat model (SD) and LPS cell model (BV2)	↓p38 MAPK, JNK, NF-κB, iNOS, IL-1β and COX-2	JUNG et al. (2013)
Phenols	anisalcohol	LPS cell model (BV2)	↓p-JNK, NF-κB, TNF-α, IL-1β, PGE2 and CD16/32	XIANG et al. (2018)
			↑TGF-β, IL-10 and CD206	
Glycosides	Total saponins of Panax japonicus	LPS cell model (BV2)	↓p-p38 MAPK, p-NF-κB, IL-1β and IL-6	WANG et al. (2022a)
	Astragaloside IV	LPS cell model (BV2)	↑p-ERK, NRF2, NO	LI et al. (2018)
	Salidroside	LPS cell model (BV2)	↓p-p38 MAPK, p-JNK, p-ERK1/2 and IL-8	HU et al. (2014)
Phenols	6-Shogaol	2VO mice model (C57BL/6) and LPS cell model (BV2)	↓p38 MAPK, JNK, iNOS, COX-2, TNF-α and IL-6	HA et al. (2012)

### 4.6 Other signaling pathway

#### 4.6.1 PPARγ signaling pathway

Peroxisome proliferator-activated receptor γ (PPARγ), a member of the nuclear hormone receptor superfamily, plays a crucial role as a transcription factor in the regulation of inflammation. Its expression is prevalent in microglia, where it contributes to the orchestration of microglia polarization, suppression of inflammation in cerebral ischemic injury, and facilitation of tissue repair (SHAN et al., 2018; LI J. et al., 2020). *Muscone*, the primary active constituent of *musk*, has been extensively employed in the management of cerebral ischemia and reperfusion injury (HAN et al., 2022). Muscone exhibits the ability to enhance the expression of Arg1 and CD206 via the PPARγ pathway, thereby significantly augmenting the conversion of microglia into M2 phenotype (REN et al., 2022; LIU et al., 2023). Additionally, it diminishes the levels of TNF-α, IL-1β, and IL-6, while up-regulating CXCL1, TGF-β, and IL-10 (REN et al., 2022; LIU et al., 2023). These effects contribute to the mitigation of inflammation and facilitation of nerve recovery at the site of cerebral infarction. *Ginkgetin*, a flavonoid obtained from *Ginkgo biloba*, can activate the PPARγ signaling pathway, enhance the expression of Arg1, IL-4, and IL-10, reduce the expression of iNOS, IL-1β, and TNF-α, and facilitate the M2 polarization of microglia. Consequently, it inhibits neuroinflammation and facilitates the restoration of neural function in ischemic brain tissue (TANG et al., 2022). Furthermore, empirical research has demonstrated that the administration of Radix Astragali IV not only induces the activation of the PPARγ pathway but also elicits a downregulation in the expression of CD86, iNOS, TNF-α, IL-1β, and IL-6, while concurrently up-regulating the expression of CD206, Arg 1, YM-1/2, IL-10, and TGF-β. This multifaceted effect facilitates the transformation of microglia/stroma phenotypes from M1 to M2, ultimately mitigating inflammation and fostering tissue regeneration (LI L. et al., 2021).

#### 4.6.2 RhoA/ROCK signaling pathway

The Ras homologous gene family member A (RhoA)/ROC kinase (ROCK) signaling pathway plays a crucial role in modulating the activities of glial cells and immune cells. Prior research has demonstrated that the inhibition of RhoA/ROCK pathway activation can lead to enhanced inflammatory response, regulation of microglia polarization, and facilitation of brain recovery (KOCH et al., 2018; LU et al., 2023). *Parthenolide*, the primary constituent extracted from *Tanacetum parthenium*, exhibits notable anti-inflammatory, antithrombotic, and neuroprotective properties (DING et al., 2022). In their study, Zhang YH et al. (ZHANG et al., 2022b) discovered that Parthenolide exhibits the ability to decrease the phosphorylation of NF-κB through the inhibition of the RhoA/ROCK signaling pathway. Additionally, Parthenolide activates the transition of microglia from M1 to M2 type, enhances the release of TGF-β, and reduces the expression of IL-1β, IL-6, and TNF-α in both *in vivo* and *in vitro* experiments (ZHANG et al., 2022b). These findings suggest that Parthenolide may possess neuroprotective properties against inflammatory responses. Liu et al. (ZHANG et al., 2016) demonstrated that *Pseudoginsenoside-F11 (PF11)*, a saponin present in *American ginseng*, exhibits neuroprotective properties against cerebral ischemic injury. The authors observed that PF11 activates the RhoA/ROCK pathway, thereby reducing the release of pro-inflammatory factors in microglia induced by oxygen-glucose deprivation (OGD). Additionally, PF11 enhances microglia phagocytosis and attenuates cerebral ischemic injury through the involvement of complement receptor 3.

#### 4.6.3 BDNF/TrkB signaling pathway

The interaction between brain-derived neurotrophic factor (BDNF) and its receptor tyrosine-induced receptor B (TrkB) plays a crucial role in the growth and maturation of neurons, thereby contributing significantly to the preservation and restoration of neuronal function (WURZELMANN et al., 2017; HABTEMARIAM, 2018). Extensive evidence supports the involvement of both BDNF and microglia activation in the development of IS, and the upregulation of BDNF and TrkB expression has been shown to effectively mitigate the accumulation of TNF-α in microglia and attenuate neuronal damage following cerebral ischemia (CHEN et al., 2015; HABTEMARIAM, 2018; INFANTINO et al., 2022). *Calycosin*, an active constituent found in *Radix Astragali*, has been extensively employed in the management of cerebral ischemia (FU et al., 2014). Research has demonstrated that Calycosin possesses the ability to diminish the population of microglia containing TNF-α through the activation of the BDNF/TrkB signaling pathway, thereby mitigating inflammation and neuronal impairment (HSU et al., 2020).

#### 4.6.4 PI3K/Akt signaling pathway

The signaling pathway of Phosphatidylinositol 3 kinase/protein kinase B (PI3K/Akt) exerts influence on various cellular processes such as cell proliferation, growth, differentiation, glucose transport, and nerve cell repair in the context of cerebral ischemic injury (WU et al., 2018; ZHONG et al., 2022). Research has demonstrated that PI3K/Akt activation triggers microglia inflammation through the activation of NF-κB. Consequently, inhibiting this pathway has been shown to effectively enhance the suppression of inflammatory mediators released during cerebral ischemic injury. *Fraxetin*, a *coumarin* compound obtained from *Fraxinus rhynchophylla*, exhibits antioxidant and anti-inflammatory properties (XU et al., 2021). Deng et al. (DENG et al., 2022) discovered that Fraxetin effectively suppresses the PI3K/Akt signaling pathway, decreases the phosphorylation of NF-κB, and diminishes the expression of TNF-α, IL-1β, and IL-6 in LPS-interfered mouse primary microglia. Consequently, it demonstrates the potential to ameliorate the activation and inflammatory response of microglia in the ischemic penumbra.

#### 4.6.5 Shh signaling pathway

The Sonic hedgehog (Shh) signaling pathway is composed primarily of the ligands Shh, Ptc, and Smo transmembrane protein receptors, along with the transcription factors Gli (GLI-1, GLI-2, and GLI-3) (YU et al., 2021). Extensive research has provided evidence that the activation of the Shh signaling pathway holds considerable potential in promoting nerve injury recovery, mitigating the activation of microglia, and attenuating a series of inflammatory responses in the context of cerebral ischemic injury (YU et al., 2021; HUI et al., 2022). *Resveratrol(Res)*, a potent constituent found in various Chinese medicinal plants including Polygonum *cuspidatum*, *cassia cuspidatum*, and *mulberry*, has been scientifically demonstrated to possess a robust neuroprotective efficacy (HE et al., 2022; GUO et al.). Liao et al. (LIAO, 2020)conducted experiments using Res to intervene in the MCAO/R mouse model and OGD/R N9 microglia model. They observed that the Shh signal was activated, leading to upregulation of PGC-1, Smo, and Gli-1 expressions. Additionally, the expressions of microglia signature protein (Iba1), TNF-α, and IL-1β were decreased, while the expression of IL-10 was increased significantly compared to the control group. These findings indicate that Res effectively improves microglia activation following cerebral ischemia and exhibits anti-inflammatory properties.

#### 4.6.6 STAT3 signaling pathway

**FIGURE 3:**
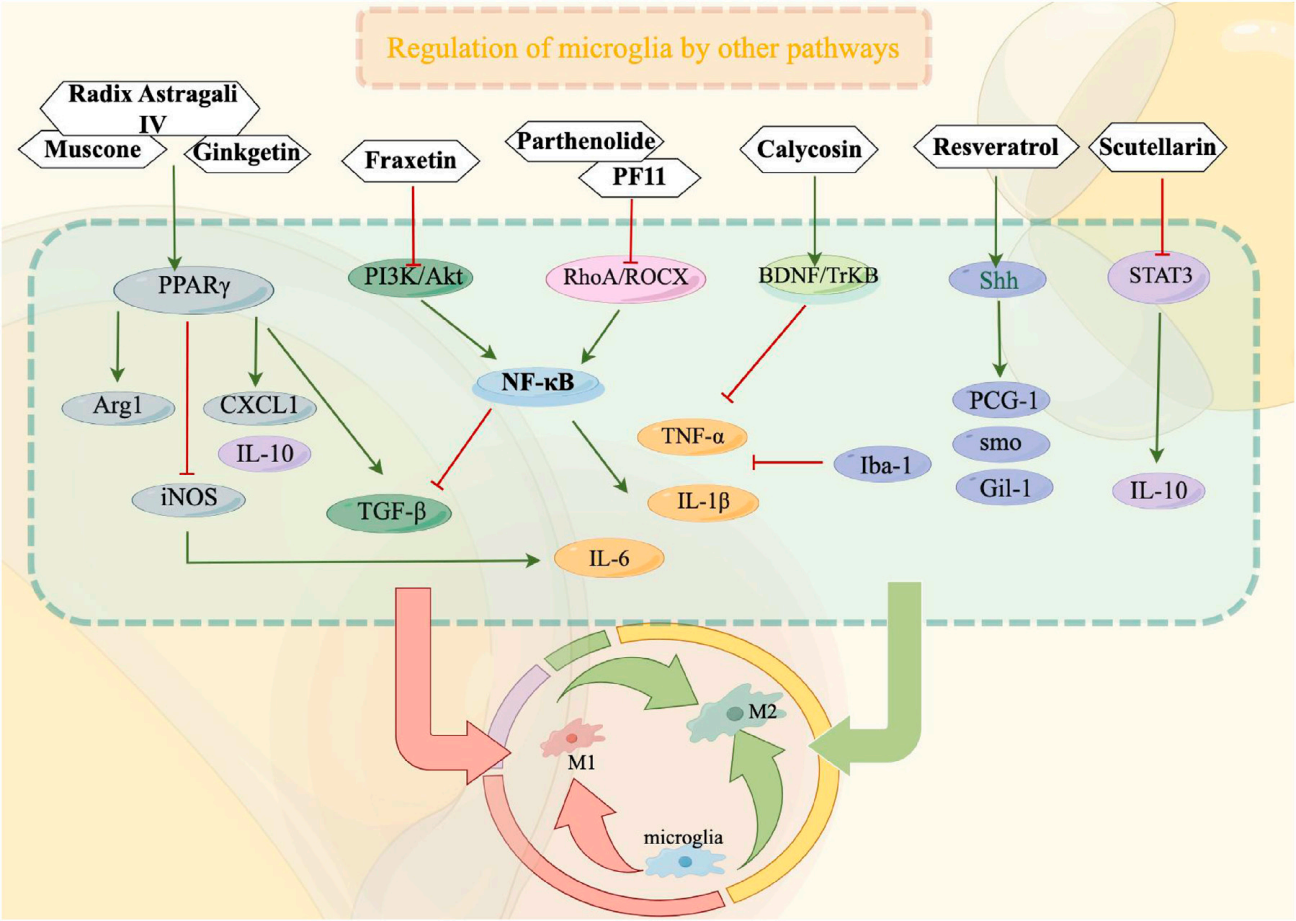
Other relevant signaling pathways play a crucial role in modulating the functioning of microglia during the treatment of ischemic stroke. By suppressing microglial activation, these pathways can effectively impede the transition from microglia to M1 phenotype or facilitate the polarization from M1 to M2 phenotype, consequently safeguarding cerebral nerves and diminishing the secretion of inflammatory mediators. In this context, red signifies inhibition, while green signifies promotion.

The signal transducer and activator of transcription (STAT) family serves as the underlying molecular mechanism for the biological activities of cytokines and plays a crucial role in the signal transduction of cytokines and growth factors (STARK et al., 2018). Comprising seven members, the STAT family includes STAT3, which is implicated in the activation of microglia and the mediation of neuroinflammatory response (STARK et al., 2018; JIA et al., 2023a). Following cerebral ischemia and reperfusion, the induction and activation of STAT3 can promote the transformation of microglia into the M1 phenotype, thereby exacerbating brain tissue injury (FANG and ZHANG, 2021). In their study, Jia et al. (JIA et al., 2023b) discovered that Scutellarin exhibited inhibitory effects on the activation of STAT3 in LPS-activated BV2 microglia. Additionally, it was observed that Scutellarin enhanced the expression of IL-10, downregulated p-STAT3, promoted the polarization of M2-type microglia, and ultimately alleviated the neuroinflammatory response associated with cerebral ischemia (Other relevant signaling pathway mechanisms are shown in Figure 3).

## 5 Drug-delivery systems

The BBB is a specialized interface between the bloodstream and brain tissue, primarily consisting of endothelial cells. These cells form tight junctions and adhesion connections that contribute to the structural integrity and isolation of the BBB. In addition to its physical barrier properties, endothelial cells are equipped with efflux transporters, such as P-glycoprotein (P-gp), which utilize ATP hydrolysis energy to actively transport foreign substances, including drugs, thereby preventing their entry into the brain parenchyma. The multi-layered structure of the BBB imposes stringent limitations on the transfer of substances between the bloodstream and the ventricle. Moreover, the distinct barriers within the BBB undergo appropriate modifications in response to physiological alterations, thereby accommodating the requirements of the central nervous system and safeguarding against disturbances in its environment. However, the distinctive physiological structure of the BBB, intricately linked to its endothelial cells, effectively shields and restricts the impact of drug molecule transportation. Numerous drug administration approaches are unable to directly traverse the BBB and reach the affected area for effective disease treatment. Consequently, the delivery strategy of TCHMs extracts across the BBB to the brain presents a significant challenge in the application of TCHMs compounds.

**FIGURE 4:**
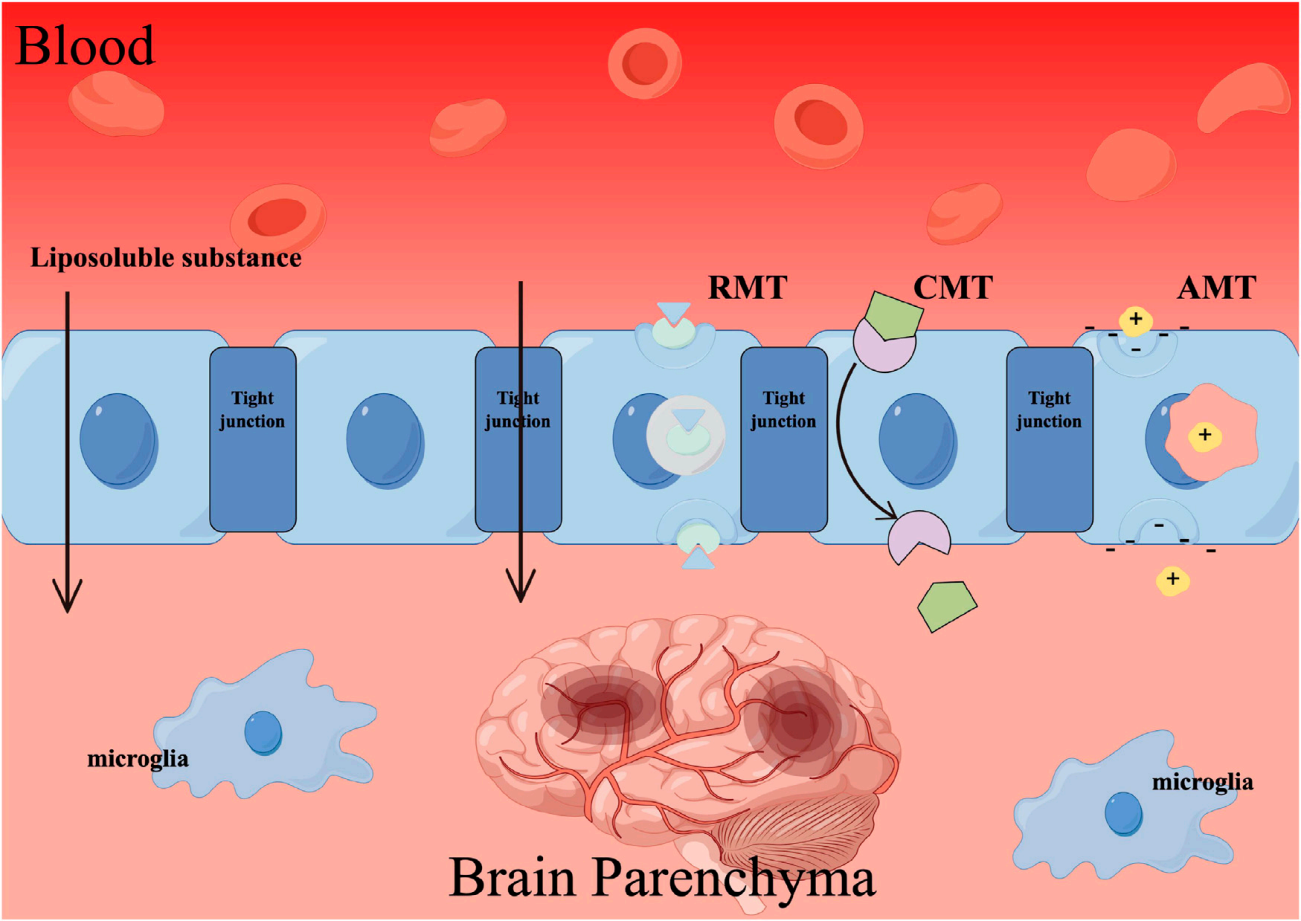
The mechanism by which traditional Chinese herbal medicines (TCHMs) traverse the BBB. Employing techniques such as receptor-mediated transcytosis (RMT), carrier-mediated transcytosis (CMT), and absorptive-mediated transcytosis (AMT) can facilitate the passage of TCHMs across the BBB, leading to a substantial enhancement in their bioavailability.

Currently, there exist primarily three approaches for achieving drug delivery to the brain through BBB targeting. The first approach is receptor-mediated transcytosis (RMT), which capitalizes on the abundance of specific endogenous receptors on the surface of the BBB. By employing ligands or antibodies as modification agents, drug delivery systems can be constructed to exploit the precise binding affinity between ligands and receptors, thereby facilitating drug penetration into the BBB via RMT. Second, carrier-mediated transcytosis (CMT) involves the binding of nutrients to transporters, which are crucial membrane proteins facilitating the absorption of various essential substances, including sugars, amino acids, lipids, vitamins, and more. The brain relies on an adequate supply of nutrients to sustain its normal physiological functions. To enable the transportation of drugs into the brain through CMT, the drug delivery system’s surface is modified with substrate analogs possessing a strong affinity for transporters. At last, absorptive-mediated transcytosis (AMT) is facilitated by the physiological attributes of BBB, particularly its negative surface charge. This characteristic enables the transportation of cationic compounds, leading to a robust electrostatic interaction between the negatively charged BBB and cationic compounds. Consequently, this interaction can be harnessed to establish an AMT mechanism through a cationic drug delivery system, thereby augmenting drug targeting within the brain (Figure 4).

### 5.1 RMT

The strategy of RMT has been extensively investigated as a prominent approach for facilitating drug delivery to the brain. Brain endothelial cells possess numerous specific receptors, including but not limited to the Transferrin (Tf) receptor, low density lipoprotein (LDL) receptor, and nicotinic acetylcholine receptor (nAChR).

#### 5.1.1 Tf

Tf has been extensively researched as a potential probe for drug delivery targeting, due to its ability to target drugs to the blood and cerebrospinal fluid barriers. The transferrin receptor (TfR) has long been a widely studied therapeutic target, as it is highly expressed in nerve cells and cerebrovascular epithelial cells (THOMSEN et al., 2022). Utilizing endogenous ligands Tf or antibodies against Tf enables drug targeting through TfR-mediated cellular uptake, effectively facilitating drug accumulation in the brain (BIEN-LY et al., 2015). Through the preparation of Tf-curcumin-longcirculatingliposomes (Tf-Cur-LCL), Ren (REN, 2019) discovered that it significantly enhanced the permeability of Cur across the BBB, resulting in a transmission rate of 37.34%. This augmentation facilitated the accumulation of therapeutic drugs in the brain, thereby achieving the desired therapeutic effect. Additionally, Tf-Cur-LCL exhibited superior protective properties towards PC-12 cells.

##### 5.1.1.1 Monoclonal antibody against TfR

Elevated endogenous levels of Tf result in near saturation of the TfR, thereby restricting the binding capacity of exogenous transferrin and diminishing its targeting efficacy (THOM et al., 2018). Monoclonal antibodies against TfR and Tf possess unique binding sites within endothelial cells and do not disrupt the interaction with natural ligands, rendering them highly utilized in drug delivery systems (HAQQANI et al., 2018). Presently, the most advanced monoclonal antibody designed to target mouse TfR is OX26 (HAQQANI et al., 2018). In their study, (SHEN et al., 2017). Employed ginsenoside Rg1 as a therapeutic agent, poly-γ-glutamic acid as a carrier, and modified the surface with OX26 antibody to fabricate nanoparticles capable of carrying the drug (referred to as PHRO). The utilization of PHRO resulted in enhanced distribution of ginsenoside Rg1 across BBB, facilitated migration and angiogenesis of RBE4 cells in rats with cerebral ischemia, reduced the volume of cerebral infarction, improved necrotic tissue, and promoted nerve injury repair. (LIU et al., 2015b). Employed the emulsification evaporation-low temperature curing method to prepare baicalin polyglycol cationic solid lipid nanoparticles modified with OX26 antibody. This modification resulted in notable enhancements in the Area Under Curve (AUC), peak time (Tmax), peak concentration (Cmax), and bioavailability. Additionally, the binding of OX26 antibody to Tf on endothelial cells facilitated the transmembrane transport of baicalin across BBB, enabling effective drug delivery.

##### 5.1.1.2 T7 peptide

T7, a peptide that specifically targets transferrin receptor (TfR), has been extensively employed for the functionalization of nanocapsules (LIANG et al., 2018). Li (LI, 2018)utilized PAMAM dendrimer as a carrier for the matrix drug, which was bound to tanshinone nanoparticles coated with bifunctional polyethylene glycol (NHS-PEG-MAL) modified with the targeting ligand T7 peptide. The findings demonstrated a notable enhancement in the pharmacological activity of tanshinone, along with a favorable brain targeting effect. Moreover, it effectively ameliorated neurological damage resulting from ischemia, reduced the extent of cerebellar infarction, and augmented the protective impact on focal cerebral ischemia-reperfusion injury.

#### 5.1.2 LDL receptor family

The LDL receptor family encompasses a broad spectrum of ligands that facilitate the transportation across cellular membranes via interactions between receptors and ligands. Prominent examples of these ligands include Lactoferrin (Lf), Angiopep-2 (ANG), apolipoprotein E (ApoE), and Polysorbate 80 (Polysorbate 80).

##### 5.1.2.1 Lf

Lactoferrin (Lf), a cationic glycoprotein belonging to the transferrin (Tf) family, exhibits natural iron-binding properties and is known to have high expression of receptors on brain endothelial cells and neurons (GRUDEN and POKLAR ULRIH, 2021). In a study conducted by NikitaKatila et al. (KATILA et al., 2022), Lf was utilized to suppress the expression of inflammatory factors induced by ischemia-reperfusion, thereby mitigating cerebral ischemic injury and demonstrating potent neuroprotective effects. Furthermore, the investigators observed that the utilization of transferrin modified polyethylene glycol-polylactic acid (PEG-PLA) nanoparticles, containing resveratrol (Tf-PEG-PLA-RSV), exhibited a noteworthy enhancement in drug accumulation within the brain when contrasted with the administration of unbound resveratrol (GUO et al., 2013; WU et al., 2020). Conducted a study in which they made modifications to the lipids-polymer hybrid nanosystem (CⅣa/LF-LPNS) that contained total saponins of P. japonicum by incorporating Lf. This modification resulted in an effective drug delivery system to the brain, leading to gradual and continuous improvement in cerebral infarction. Additionally, it significantly enhanced the survival rate of rats and resulted in a peak drug concentration in the brain that was five times higher than that observed in the total saponins solution group of P. japonicum. Furthermore, the Lf-modified quercetin and astragaloside IV liposomes demonstrated notable permeability across the BBB and exhibited the ability to prevent neuronal loss (KUO and TSAO, 2017; YANG, 2022).

##### 5.1.2.2 ANG

The ANG peptide consists of 19 amino acids and serves as a ligand for the low-density lipoprotein receptor related protein 1 (LRP-1) receptor, specifically targeting the BBB. The Ang-modified nanoparticles exhibit a strong affinity for LRP-1 and demonstrate notable brain targeting capabilities without compromising the integrity of the BBB (OLLER-SALVIA et al., 2016). Research findings indicate that ANG-modified salidroside liposomes exhibit a heightened drug encapsulation rate, effectively enhancing drug uptake in the brain, and addressing the issue of inadequate drug delivery to the lesion area. Additionally, these liposomes prolong drug efficacy duration and sustain drug concentration stability at the site of the lesion (ZHANG et al., 2022c). Furthermore, the efficacy of Iicariin liposomes is similarly enhanced when modified by ANG (ZHANG et al., 2020c).

##### 5.1.2.3 ApoE

ApoE exhibits robust expression in cerebral vascular endothelial cells and possesses the ability to selectively interact with LDL receptors and LRP1 receptors located on brain endothelial cells, thereby facilitating its transportation across the BBB via endocytosis (DAL MAGRO et al., 2017). Through the utilization of a carboxyl functionalized mesoporous silica nanocarrier, researchers successfully coated berberine with ApoE, leading to noteworthy outcomes. Comparative analysis with the control group revealed that berberine exhibited a substantial increase in both average drug retention time and half-life, while concurrently demonstrating a reduction in vivo clearance rate (ZHANG et al., 2020c). Moreover, the brain targeting index (DTI) surpassed the value of 1, indicating enhanced brain-specific accumulation of berberine.

##### 5.1.2.4 Polysorbate 80

Polysorbate 80, a non-ionic surfactant, exhibits affinity for very low-density lipoprotein receptors on brain endothelial cells, enabling it to traverse the BBB (SINHA et al., 2020). Consequently, it is frequently employed as a coating agent to facilitate receptor-mediated endocytosis into the brain. When Puerarin is encapsulated within Polysorbate 80-coated poly (lactate-co-glycolic acid) (PLGA) nanoparticles, it demonstrates enhanced pharmacokinetic parameters, including a greater area under the curve (AUC), mean residence time (MRT), half-life (t1/2), and reduced plasma clearance (CL). Polysorbate 80 is utilized to create a hydrophilic layer on the surface of nanoparticles (NP), which serves to impede electrostatic and hydrophobic interactions (SUN et al., 2015). This layer also prevents Opsonin protein absorption, thereby reducing phagocytic action and prolonging the presence of nanoparticles in the bloodstream, furthermore, Polysorbate 80 inhibits the efflux of P-gp from brain endothelial cells, thereby enhancing drug distribution in the brain (SUN et al., 2015; TAO et al., 2021). Furthermore, (TIAN et al., 2018). Developed a modified curcumin-loaded hyaluronic acid-β-curcumin conjugate, known as Cur-HSC, which was modified with Polysorbate 80. This modification resulted in a micellar AUC that was approximately 4.70 times greater than that of free curcumin when administered intravenously. Moreover, curcumin demonstrated effective accumulation in the brain.

#### 5.1.3 nAChR

nAChR can be expressed in brain capillary endothelial cells (DURIS et al., 2017). Polypeptide fragments of the rabies virus glycoprotein (RVG) are known for their ability to permeate the BBB and their non-invasive nature (DOS SANTOS RODRIGUES et al., 2020a). Currently, mature derived peptides such as RDP and RVG-29 are utilized for targeted drug delivery by binding to nAChR (HUEY et al., 2017a; HUEY et al., 2017b).

##### 5.1.3.1 RVG-29 peptid

RVG-29, a 29-amino acid sequence polypeptide within RVG, exhibits specific binding affinity to nAChR (ZHOU et al., 2022). Through its interaction with nAChR, RVG-29 facilitates the transportation of drugs into brain endothelial cells, across the BBB, and ultimately into the brain (HAO et al., 2020; ZHOU et al., 2022). Consequently, RVG-29 has been extensively employed in nanocarriers to enhance brain-targeting capabilities. (HAN et al., 2020). Employed erythrocyte membrane-coated nanostructured lipid particles (NPs @ RBCm) in conjunction with RVG29 for the purpose of facilitating the transportation of resveratrol across the BBB and its subsequent accumulation within neurons. Both *in vitro* and *in vivo* experiments have demonstrated that this delivery system exhibits not only favorable safety characteristics, but also superior BBB permeability when compared to free resveratrol, while additionally exhibiting the ability to selectively bind to specific neurons.

##### 5.1.3.2 RDP peptide

(ZHAO et al., 2018). Employed RDP-modified nanoliposomes as a delivery vehicle for curcumin with the aim of targeting the brain. The utilization of curcumin RDP liposomes (RCL) resulted in notable enhancements in brain targeting, water solubility, and biocompatibility of the drug when compared to free curcumin.

### 5.2 CMT

Endogenous nutrient transporters on the BBB, such as glucose transporter 1 (GLUT1) and macromolecular neutral amino acid transporter 1 (LAT1), have emerged as effective targets for facilitating drug penetration into brain tissue.

#### 5.2.1 Glucose transporters

Glucose transporters facilitate the transportation of glucose and other hexoses, with GLUT1 being identified as the most proficient transport system across the BBB (ANRAKU et al., 2017). Research has demonstrated that the glycosylation-modified drug delivery system can be selectively acknowledged and transported by GLUT1 (FU et al., 2019; WANG et al., 2022b). Conducted a study wherein they devised and synthesized two variations of glycosylated quercetin, namely, GLU-Que (modified with a single glucose group) and 2GLU-Que (modified with two glucose groups). The incorporation of a sugar group has been found to have a substantial impact on the water solubility and bioavailability of quercetin. Glycosylation has the potential to enhance quercetin’s ability to target the brain, elevate its drug concentration, and consequently result in heightened neuroprotective effects. Furthermore, it has been observed that 2Glu-Que exhibits a greater neuroprotective capacity compared to Glu-Que, as evidenced by its ability to reduce the ischemic area to 5.06%.

P-aminophenyl-α-D-mannopyranoside (MAN) is a structurally similar compound to mannose. MAN exhibits a specific affinity for GLUT1, facilitating its transportation. Extensive research has demonstrated that liposomes modified with MAN not only enhance drug permeation across the BBB, but also enable targeted drug release within specific brain regions (HAO et al., 2013; NAVONE et al., 2013). *In vitro* experiments have revealed that MAN-modified liposomes containing curcumin and quinacrine significantly enhance the bioavailability of curcumin, surpassing the efficacy of free curcumin in BBB penetration (WANG Y. et al., 2017).

#### 5.2.2 LAT

The LAT1 transporter, functioning as a heterodimer, exhibits the capability to transport various amino acids such as leucine, phenylalanine, tyrosine, tryptophan, histidine, etc., in a Na + -independent manner with notable affinity (KANAI, 2022). Termed as a neutral and branched-chain amino acid exchanger, it predominantly resides within the brain capillary endothelial cells and neurons. Consequently, it has been employed as a prodrug for facilitating the delivery of central nervous system drugs via a transport mechanism resembling that of its substrates (GARCIA et al., 2023). The researchers employed LAT1 as a means to synthesize prodrugs that facilitate the direct binding of ferulic acid to the side chain of 1-phenylalanine via amide or ester bonds. Both prodrugs exhibited superior penetration rates compared to ferulic acid. *In vivo* and *in vitro* experiments demonstrated the effective utilization of LAT1 to enhance the pharmacokinetics of the central nervous system in mice, resulting in a significant increase in the AUC of brain tissue by up to 32-fold and an elevation of the Cmax from 3.6 μM to 13.1 μM (PURIS et al., 2019).

### 5.3 AMT

The negatively charged surface of the BBB is a result of the polarized distribution of sialic acid on the luminal side and heparin sulfate on the basement membrane side (SANTA-MARIA et al., 2019). This charge allows for electrostatic adsorption, enabling certain cationic molecules to bind to surface anion sites. The anionic sites on the cavity side facilitate interaction with cationic substances, leading to adsorption-mediated endocytosis (MUNISWAMY et al., 2019; SANTA-MARIA et al., 2019). Additionally, the anion sites on the basement membrane side promote the externalization of cationic substances from the lumen to the cerebral interstitium (SANTA-MARIA et al., 2019; YU et al., 2019). The present study demonstrates that the presence of a positively charged drug delivery system on the surface can effectively initiate adsorption-mediated transcytosis across the BBB, thereby facilitating drug delivery into the brain (YU et al., 2019). Notably, basic polypeptides and cationic proteins, including albumin, histones, and avidin, can be employed in conjunction with drugs or drug delivery systems to facilitate drug transportation into the brain via electrostatic adsorption (MENDES et al., 2018; MUNISWAMY et al., 2019; YU et al., 2019). Specifically, cationic albumin has gained significant prominence as a modification agent for brain-targeted drug carriers.

(YE et al., 2021). Employed cationic liposomes possessing robust biological adhesion as carriers to fabricate curcumin cationic liposomes via the ethanol injection technique. These liposomes exhibited therapeutic efficacy by facilitating the transportation of drugs into the brain through mediation with cationic liposomes. Additionally, they interacted with negatively charged nasal mucosa, thereby prolonging the retention duration of liposomes within the nasal cavity. The AUC of curcumin in brain tissue exhibited a 1.19-fold increase, while the clearance rate in brain tissue experienced a reduction of 1.59%. These alterations aim to enhance drug targeting within the brain, augment bioavailability, and prolong elimination kinetics. Consequently, this interaction enhanced the drug’s capacity to traverse the cell membrane via adsorption-mediated endocytosis, resulting in efficient transport and absorption of the drug across the BBB. The researchers prepared Baicalin PEGylated cationic solid lipid nanoparticles, modified with the OX26 antibody, were synthesized using the emulsification evaporation-low temperature curing method. *In vitro* and *in vivo* experiments revealed that the positively charged baicalin solid liposome nanoparticles exhibited enhanced adsorption on the negatively charged cell membrane, resulting in higher values for AUC, Tmax, Cmax, and bioavailability in cerebrospinal fluid compared to free drugs (LIU et al., 2015b).

Furthermore, a majority of cell penetrating peptides (CPPs) possess a positive charge and undergo surface modifications on the carrier in order to facilitate drug delivery into the brain via adsorption-mediated transcytosis. By virtue of their cell membrane penetration capability, CPPs effectively augment drug accumulation within the brain (TAYLOR and ZAHID, 2020). R8, a cationic transmembrane peptide, exhibits enhanced electrostatic interaction with liposomes and nasal mucus following modification, thereby prolonging its retention time in the nasal cavity (KAMEI et al., 2018; DOS SANTOS RODRIGUES et al., 2020b). Research findings indicate that the co-administration of R8 and β-asarone with resveratrol nanoparticles via nasal administration effectively elevates drug concentration in the brain, surpassing the concentration observed in the liver and kidney. This approach facilitates sustained drug release while minimizing potential damage to the liver, kidney, and other organs (YU, 2019).

## 6 Conclusion and prospect

**FIGURE 5:**
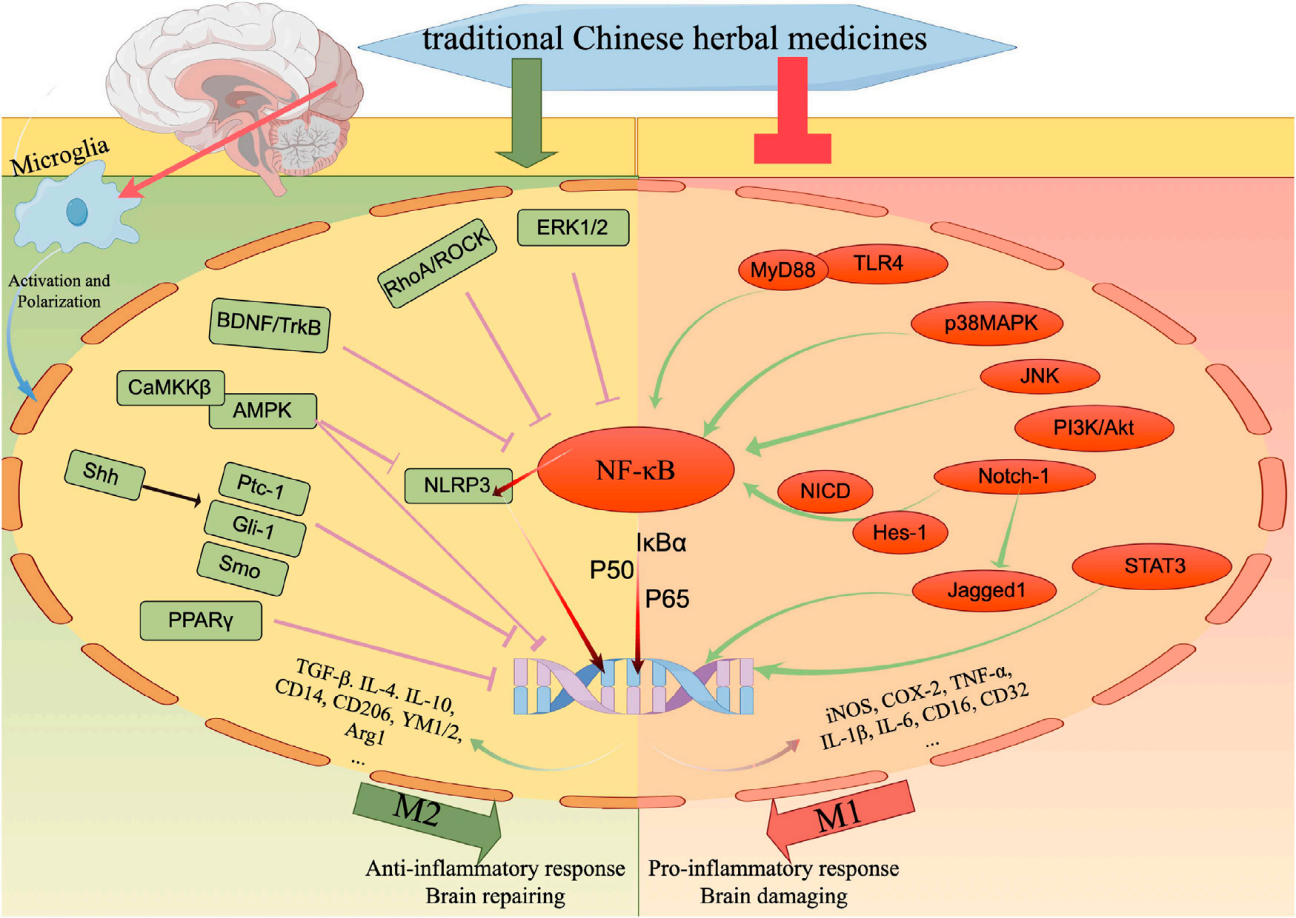
The regulatory effect of TCHMs on microglial response to cerebral ischemia. After cerebral ischemia and reperfusion, microglia immediately sense the disorder of brain homeostasis, and are activated and polarized. The M1 phenotype of polarized microglia is associated with the pro-inflammatory response of microglia to cerebral ischemic injury, while the M2 phenotype promotes anti-inflammatory response and brain repair. TCHMs can regulate these microglial responses by inhibiting M1 phenotype-related signaling pathways and molecular targets and enhancing M2 phenotype-related pathways, thereby reducing cerebral ischemic injury and promoting nervous system repairation.

Neuroinflammation is a crucial factor in the development of IS. Microglia, as the primary immune cells responsible for protecting the brain against injury, undergo morphological and functional alterations in response to danger signals triggered by IS. Consequently, the inflammatory response regulated by the activation of microglia has garnered significant attention in the treatment of IS. However, the dynamic response of microglia to cerebral ischemia and reperfusion injury undergoes a transition from the initial pro-inflammatory M1 phenotype to the subsequent anti-inflammatory M2 phenotype. These contrasting effects of microglia suggest that the impact of TCHMs on M1 and M2 phenotypic alterations at varying time points following IS holds significant value and feasibility. A substantial body of experimental evidence demonstrates that TCHMs possess the ability to mitigate neuroinflammation by modulating microglia. The specific mechanism is illustrated in Figure 5. The primary signaling pathways implicated in this study are NF-κB, TLR4, and MAPK. Notably, TCHMs exert their inhibitory effects by disrupting the interaction between TLR4 and MyD88 in microglia, prevent the formation of complexes to decrease NF-κB and p38, ERK1/2 phosphorylation. Consequently, this inhibition leads to a reduction in M1 microglia and an increase in M2 polarization. Alternatively, TCHMs can also inhibit the p38 MAPK signaling pathway, downregulate the phosphorylation of NF-κB, decrease the release of inflammatory mediators, and impact microglial activation. Furthermore, it has the ability to modulate the polarization of microglia from M1 to M2 through the direct inhibition of the NF-κB signaling pathway, thereby ameliorating the inflammatory response and safeguarding neurons. On one hand, numerous *in vitro* and *in vivo* studies have demonstrated the efficacy of traditional Chinese herbal medicines (TCHMs) in regulating microglial cells for the treatment of IS. On the other hand, a considerable body of literature has explored the anti-neuroinflammatory properties of TCHMs through the modulation of various pathways. However, further research is necessary to investigate TCHMs as multi-target neuroprotective agents, particularly through clinical studies of IS. Moreover, these substances hold potential as primary components for the development of potent new neuroprotective agents. Drawing from the data compiled in this review, it is reasonable to hypothesize that certain impacts are direct while others are indirect. To enhance the efficacy of neuroprotection, a comprehensive understanding of the objectives underlying these effects is imperative. In recent years, the integration of bioinformatics and computer-aided drug discovery/design techniques has emerged as a pivotal factor in advancing the development of therapeutically significant small molecules. In light of this, we propose the utilization of bioinformatics methodologies to elucidate the precise pharmacological targets of TCHMs for neuroprotection. Subsequently, this knowledge can be leveraged to design and fabricate more potent neuroprotective agents, thereby facilitating the transition of TCHMs research from a purely scientific pursuit to a clinical practice. Altogether, additional randomized clinical trials and extensive animal models of MCAO are indispensable in order to furnish a more dependable body of evidence.

It is noteworthy that Baicalein, Luteolin, Scutellarin, and other compounds possess the advantages of multi-target and multi-pathway mechanisms, enabling them to simultaneously modulate multiple pathways and exert neuroprotective effects. Furthermore, when combined with the characteristics of different TCHMs, they can effectively counteract the neuroinflammatory response triggered by microglia following cerebral ischemic injury through various forms of crosstalk and mutual influence. This finding holds potential as a novel reference for guiding clinical medication in the treatment of IS. Given the intricate pathogenesis of IS, the combined administration of TCHMs represents a superior approach to enhance therapeutic outcomes. Simultaneously, the previous research also revealed that estrogen serves as the primary determinant for the gender disparity in the occurrence of IS. Moreover, our findings indicate that a significant proportion of the pharmaceutical compounds identified are flavonoids, which play a crucial role in various signaling pathways involved in the therapeutic management of IS. The brain endothelial cell membrane, characterized by its lipid-based bilayer structure and lipophilic nature, further supports the preferential permeability of flavonoids, being fat-soluble drugs, across the BBB compared to their water-soluble counterparts. Consequently, a substantial body of *in vivo* and *in vitro* investigations have centered on the examination of flavonoids as the primary research subject, owing to their enhanced efficacy in the treatment of IS. Following menopause, women experience a substantial elevation in the susceptibility to IS; however, estrogen-like TCHMs exhibit the ability to not only impede neuroinflammation by selectively targeting the NF-κB signaling pathway in BV2 microglia but also exert a notable neuroprotective effect (EL-BAKOUSH and OLAJIDE, 2018). Consequently, the utilization of natural botanical remedies such as Genistein, Daidzein, and other alternative hormone therapies for postmenopausal IS emerges as an efficacious approach.

Our focus extends to the extraction and bioavailability of natural plant drugs. The inherent physical characteristics of TCHMs pose challenges in terms of purification and purity. Furthermore, the bioavailability of TCHMs *in vivo* is hindered by the first-pass effect, resulting in limited absorption into the bloodstream and subsequent utilization by the body.

Hence, contemporary research is primarily concerned with the advancement of brain-targeted delivery systems that exhibit a high utilization rate, enabling drugs to traverse the BBB and reach the site of injury. Among the various transport mechanisms employed for brain targeted TCHMs, RMT has garnered significant attention and application. By leveraging the distinctive properties of specific receptors within the BBB, TCHMs are encapsulated within corresponding ligands, facilitating receptor-ligand binding and facilitating drug transport across the BBB. Simultaneously, it is worth acknowledging that the aforementioned TCHMs are predominantly administered through injection, oral ingestion, and nasal delivery. The TCHMs’ bioavailability is notably diminished due to inherent drug properties as well as hepatic and intestinal metabolism. Nevertheless, nasal administration offers advantageous attributes. It is convenient mode of delivery, abundant vasculature, swift absorption, evasion of the first-pass effect, enhanced TCHMs bioavailability, and evident brain targeting collectively exert a substantial positive impact on the treatment of IS. Furthermore, the utilization of flavonoids and their delivery systems, known for their antioxidation, anti-inflammation, and anti-apoptosis properties, has been extensively employed in the treatment of IS. Flavonoids possess the ability to readily penetrate the brain when coupled with various ligands such as Tf, T7, and Lf, facilitating the continuous and efficient release of therapeutic agents. This phenomenon has been substantiated through numerous *in vitro* and *in vivo* experiments. Nevertheless, it is important to acknowledge that the RMT approach also presents certain limitations, as the receptor is not solely present in the BBB but is also expressed in the capillaries of other peripheral organs. Whereas, while attaining brain targeting, it augments drug accumulation in peripheral tissues, necessitating thoughtful consideration in selecting appropriate ligands. Furthermore, we underscore the limitations associated with nasal administration, encompassing the presence of nasal fibrous hair that facilitates drug elimination, thereby necessitating strategies to prolong drug retention in the nasal cavity and enhance drug absorption. Additionally, nasal administration imposes constraints on the physical and chemical attributes as well as particle size of the drugs. At all, the integration of effective extraction techniques with sophisticated delivery systems to facilitate the selective passage of TCHMs across the BBB and their subsequent therapeutic efficacy represents not only the focal point of TCHMs’ advancement and refinement but also a formidable obstacle for their prospective clinical implementation.

Furthermore, it has been observed that numerous investigations have employed isolated primary microglia or microglia-like cell lines for *in vitro* experimentation. While these studies offer insights into the correlation between drugs and microglia, they fail to consider the intricate brain environment and the interplay between neurons and astrocytes. Consequently, future research should incorporate more *in vivo* experiments or co-culture systems to substantiate the reciprocal communication between these entities, thereby facilitating the advancement of novel therapies for IS.
